# Altered Intrinsic Brain Activity in Patients With Toothache Using the Percent Amplitude of a Fluctuation Method: A Resting-State fMRI Study

**DOI:** 10.3389/fneur.2022.934501

**Published:** 2022-06-23

**Authors:** Jun Yang, Yi Shao, Yan-Kun Shen, Hong-Shui Zhu, Bin Li, Qiu-Yue Yu, Min Kang, San-Hua Xu, Ping Ying, Qian Ling, Jie Zou, Hong Wei, Yu-Lin He

**Affiliations:** ^1^The Affiliated Stomatological Hospital of Nanchang University, The Key Laboratory of Oral Biomedicine, Nanchang, China; ^2^Department of Ophthalmology, The First Affiliated Hospital of Nanchang University, Nanchang, China; ^3^Department of Radiology, The First Affiliated Hospital of Nanchang University, Nanchang, China

**Keywords:** toothache, percent amplitude of fluctuation, support vector machine, receiver operating characteristic, pathological mechanism

## Abstract

**Objective:**

The percent amplitude of fluctuation (PerAF) technique was utilized to evaluate the neural functions of specific cerebrum areas in patients with toothache (TA).

**Patients and Methods:**

An aggregation of 18 patients with TA (eight males and 10 females) were included in the study. We also recruited 18 healthy controls (HCs; eight men and 10 women) aligned for sex and age. Resting functional magnetic resonance imaging (rs-fMRI) scans were obtained. Then, we utilized the PerAF method and a support vector machine (SVM) to analyze the image data and measure neural abnormalities in related cerebrum areas. Receiver operating characteristic (ROC) curve analysis was utilized to appraise the two data sets.

**Results:**

The PerAF signals in the right dorsolateral superior frontal gyrus (RDSFG) and the right posterior central gyrus (RPCG) of TA sufferers were lower than HC signals. These results may reveal neural dysfunctions in relevant cerebrum regions. The AUC values of PerAF in the two areas were 0.979 in the RDSFG and 0.979 in the RPCG. The SVM results suggested that PerAF could be utilized to distinguish the TA group from HCs with a sensitivity of 75.00%, a specificity of 66.67%, and an accuracy of 70.83%.

**Conclusion:**

Patients with TA had marked differences in PerAF values in some regions of the cerebrum. Changes in PerAF values represented distinctions in blood oxygen level dependent semaphore intensity, which reflected the overactivity or inactivation of some cerebrum areas in those suffering from TA. At the same time, we analyzed the PerAF values of TAs with ROC curve, which can be helpful for the diagnosis of TA severity and subsequent treatment. Our results may help to elucidate the pathological mechanism of TA.

## Introduction

Toothache (TA) is a common oral clinical symptom caused by various stimuli to the tooth itself and its surrounding soft tissues. It can be caused by multiply diseases such as dental caries, pulpitis, periapical periodontitis, gingivitis, periodontitis, and oral ulcers. Due to its innervated nature, pain is the only way the pulp and dentin respond to various external stimuli and the pain manifests as persistent, dull, throbbing, and radiating in a variety of ways (1). Dental pulp is a tissue with high nerve density, it is rich in sensory nerve endings, and it plays a crucial role in regulating odontogenic tooth pain, responses to external stimuli, and detection of potential tooth damage (2). Untreated pain can have physical effects such as dyspnea, ascending heart rate and blood pressure, increased myocardial oxygen demand, increased stress hormones, vomiting, and muscle tension (3). Long-term TA affects the patients' mental state and sleep quality, and even causes the patient to suffer from depression, anxiety, and other psychological conditions, often accompanied by migraines. In addition, it brings a substantial economic burden to society and individuals (4). In general, TA is more common among people of lower socioeconomic status because of their limited access to dental care. In addition, various risk factors, such as education level, smoking, and drinking habits, and race/ethnicity are associated with TA (5). TA can be an early warning mechanism. If the dental disease causing the pain is not treated in time and effectively, it can lead to severe complications, such as infection, that may be life-threatening (6). Despite the rapid progress in treatment methods, such as drugs and surgery, the mechanism of brain structure and function changes in patients with TA remains unclear. In recent years, studies have shown that TA can manifest multiple chronic pains, including paroxysmal throbbing pain in migraines, dull persistent pain in joint dysplasia, or concentrated facial pain. In addition, the degree of TA and the severity of the lesions do not change linearly, and the pain experience is closely related to personal characteristics and psychological factors. (7) Moreover, diagnosis of TA can be complicated. Therefore, exploring brain-related responses to TA has profound theoretical and clinical implications for understanding the cognitive regulation of pain systems.

At present, the use of imaging methods to probe cerebrum area configurations and functions can contribute to exploring the neural mechanisms of TA. Rs-fMRI has been recognized as an appropriate method for assessing TA-related brain damage. Regional homogeneity, low-frequency fluctuation (ALFF) amplitudes, and fractional ALFF (fALFF) amplitudes are three essential rs-fMRI approaches for handling regional cerebrum abnormalities. However, these methods are susceptible to breathing and cardiac noise artifacts and subsequent statistical analysis is complicated due to the arbitrary units of the blood oxygen level dependent (BOLD) signal (8–10). Regional homogeneity (ReHo) is a new method that was developed in recent years. ReHo breakthroughs the theoretical assumption based on linear time invariance and is a new hypothesis for brain fMRI data analysis. However, based on temporal consistency, ReHo cannot be used to analyze the functional state of the cerebrum integrally (11). In recent years, studies have proposed the application of multiple analytical methods for resting-state fMRI, suggesting that many cerebrum areas in patients with TA have abnormalities in intrinsic brain activity. The percent amplitude of fluctuation (PerAF) method can be applied to signal changes in MRI to evaluate the percentage of rs-fMRI (12). PerAF can be used to measure the percentage of BOLD fluctuations and the mean for the whole time series can be calculated. In contrast to ALFF, PerAF is a scale-independent method (13) and is similar to the percent signal change in rs-fMRI. Furthermore, PerAF prevents the confounding variables of voxel-specific fluctuation magnitudes to affect the results (14). Several studies have suggested that PerAF exhibits analogous intra-scanning reliability to ALFF, as it is less affected by BOLD signal intrusion and can be a more reliable indicator (12).

The PerAF has an excellent potential for voxel analysis. However, few investigations have been carried out to probe typical lesions in the cerebrum in patients with TA. Therefore, this study may be very helpful for inchoate studies that can be a basis for diagnosis and prevention of cerebral abnormalities in patients with TA.

## Methods and Subjects

### Subjects

Eighteen patients (eight men, 10 women) with TA were recruited for the study at two hospitals, namely, the Affiliated Stomatological Hospital of Nanchang University and the First Affiliated Hospital of Nanchang University. All subjects conformed to the following criteria: (1) no obvious malformation of the cerebrum on MRI; (2) no history of mental illness; (3) no history of drug or alcohol abuse; and (4) no claustrophobia.

The inclusion standards for the TA group were as follows: (1) pain caused by dental disease or non-dental disease in the pulp and/or periodontal tissue; (2) acute or chronic TA; (3) no other comorbid pain sickness; (4) tolerant of MRI examination; (5) routine MRI T1WI and T2WI sequence examination showing no evident abnormality; and (6) TA with no apparent cause and not attributable to other diseases. The exclusion criteria were as follows: (1) presence of non-dental pain; (2) first-degree relatives with genetic non-dental pain syndromes; (3) suffering from mental disorders; and (4) presence of contraindications for MRI scanning.

This study included 18 healthy controls (HCs; eight men, 10 women) with matched ages and sex. Inclusion criteria for HCs were as follows: (1) no TA symptoms; (2) no parenchymal malformations on MRI; (3) no mental disorder; and (4) feasible MRI examination.

### Ethical Approval and Consent to Participate

Based on the Declaration of Helsinki, this research was authorized by the medicinal morality council of Nanchang University's First Affiliated Hospital (Nanchang, China PR). Each participant signed a declaration of informed consent.

### MRI Data Acquisition

#### FMRI Data Parameters

A Trio 3-Tesla MR scanister (Siemens, Munich, Germany) was utilized for MRI scanning. All participants were directed to close their eyes and remain under spontaneous breathing through the scanning. A metamorphic gradient echo sequence was utilized to obtain the data. Then, the desired functional images were received with a 3D metamorphic gradient echo pulse sequence.

#### FMRI Data Analysis

In this study, we performed an analysis of the aforementioned functional maps. During magnetization equalization, the first 15 time points were abandoned. We utilized Rs-fMRI Advanced Edition (DPARSFA 4.0, http://rfmri.org/DPARSF) software, Rs-fMRI Data Analysis Toolkit (REST, http://www.restfmri.net), and Statistical Parameters Mapping software (SPM8, http://www.fil.ion.ucl.ac.uk/spm) for head motion rectification, dimensional normalization, and slice timing. Then, we applied Digital Imaging Communication system to smooth the data. Subjects were eliminated if they had exorbitant angular movements or a maximal excursion >1.5 mm in the x, y, or z orientation during the fMRI scanning (1). Erroneous variables in the signals from the central white matter region of the brain and the ventricular region of interest (ROI) were dispelled with linear regression. Finally, the functional graphics were normalized utilizing standard echo-plane graphic templates. We creatively utilized the PerAF technique to handle the fMRI datum. The formula for calculating the individual PerAF value is as follows:


(1) $$PerAF=\frac{1}{n}\sum {}_{i=1}^{n}\left|\frac{x_{i}-\mu }{\mu }\right|\times 100\%$$

(2) $$\mu =\frac{1}{n}\sum {}_{i=1}^{n}x_{i}$$

where x_i_ portrays the signal intensity at the ith time point, n symbolizes the totality of time points, and μ stands for the average value of the time series.

### Support Vector Machine Analysis

We applied the LIBSVM software (http://www.csie.ntu.edu.tw/~cjlin/libsvm/) to determine whether patients with TA could be differentiated by the increase or decrease in the intensity of PerAF in different brain regions and HCs. SVM was done by “leave one out.” At the same time, we validated the SVM results by running the permutation test 10,000 times. Thereby, the global accuracy of each sample could be obtained.

### Statistical Analysis

We performed independent-sample *t*-tests with statistical software (SPSS 20.0; SPSS, Chicago, IL, USA) to compare the two groups. Results were statistically significant when *P* < 0.05. Discrepancies in PerAF values between the TA group and HCs were compared with a two-sample *t*-test using the SPM8 toolkit (the statistical threshold was set at *P* < 0.05 for multiple comparisons with a Gaussian random field, and Voxel-level AlphaSim was set at *P* < 0.01 and rectified for cluster sizes >40 voxels).

The PerAF in specific cerebrum areas in the TA group and HCs were analyzed with ROC curve analysis. Pearson's correlation analysis was used to assess the connection between particular cerebrum areas and their clinical manifestations in patients with TA. SVM correlation analysis was performed to obtain and guarantee the global accuracy of each sample.

## Results

### General Situation and Clinical Data Comparisons

There were no noticeable differences in the age and sex distributions between the TA group and the HC group (*P* = 0.679 and *P* > 0.99, respectively). The disease duration of patients with TA was 2.04 ± 1.08 years. More details are summarized in Table 1.

**Table 1:** Comparison of baseline data between toothache patients and healthy subjects.

	**TA group**	**HC group**	***T*** **-value**	***P*** **-value**
Sex	8/10	8/10	N/A	>0.99
Age	41.18 ± 11.65	40.73 ± 12.48	0.088	0.679
Dominant hand	18 (right hand)	18 (right hand)	N/A	>0.99
Disease duration (years)	2.04 ± 1.08	N/A	N/A	N/A
Visual analog score	6.36 ± 1.49	N/A	N/A	N/A

*Two independent t tests were used to compare the baseline data of the two groups (P < 0.05 indicates that the difference between the two groups was statistically significant). Data are presented as mean ± standard deviation or n*.

*TA, toothache; HC, healthy control*.

### PerAF Differences

**Figure 1:**
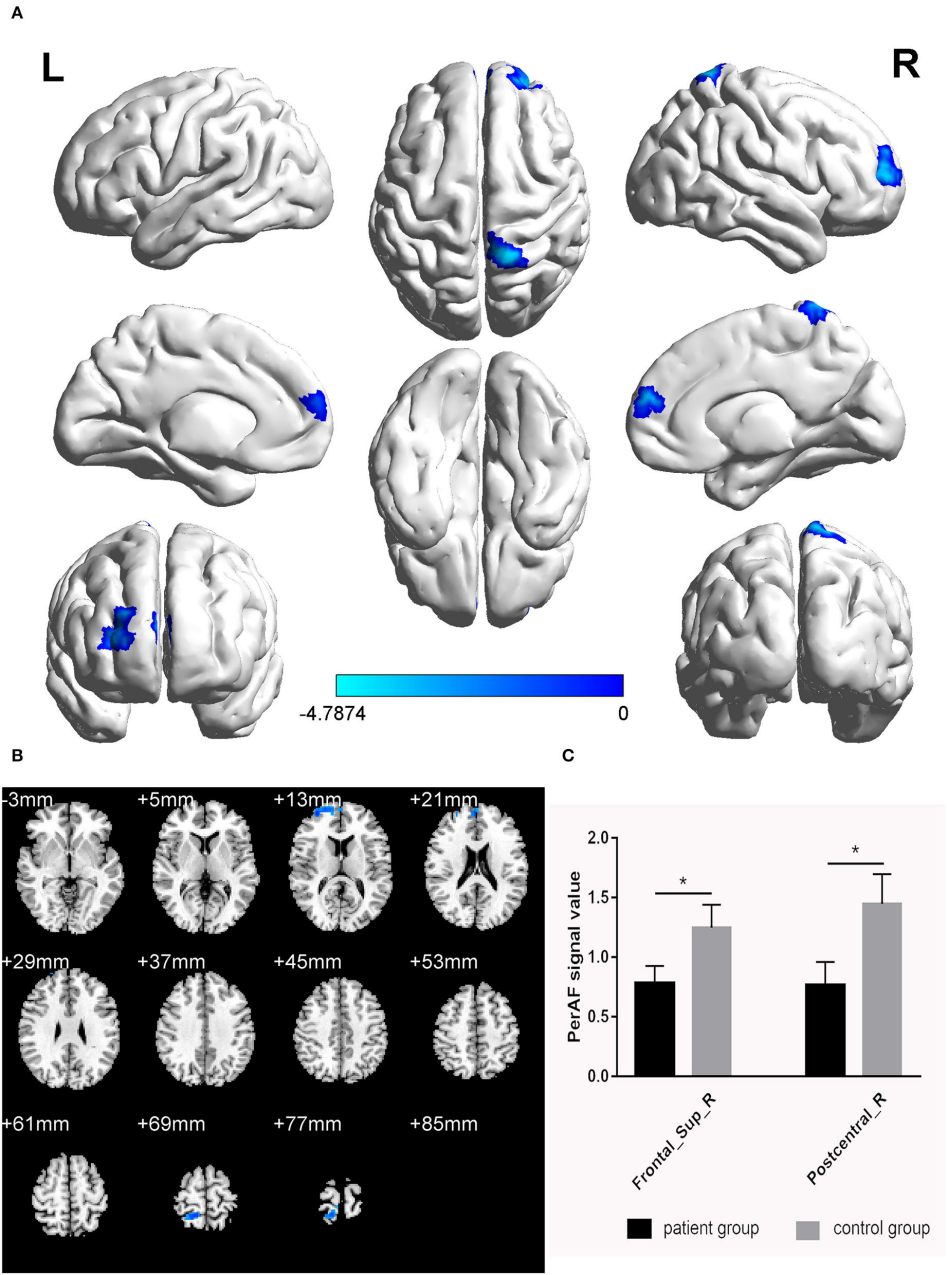
Significant differences in brain activity **(A,B)**. Blue areas indicate lower PerAF values. AlphaSim performed multiple comparisons at cluster sizes >40 voxels and *P* < 0.05, obtained by Gaussian random field theory. **(C)** ALFF mean values between TA group and HC. PerAF, percent amplitude of fluctuation; Frontal_Sup_R, right dorsolateral superior frontal gyrus; Postcentral_R, right posterior central gyrus. *represents significant difference.

The PerAF values in the cerebrum areas of the TA group, including the right dorsolateral superior frontal gyrus (RDSFG) and the right posterior central gyrus (RPCG), were decreased to varying degrees (Figures 1A,B and Table 2). The average ALFF of the two groups is shown in Figure 1C. The PerAF values of the two regions in the TA group had no correlation with clinical manifestations (*P* > 0.05).

**Table 2:** Brain regions with significant differences in PerAF between patients and HCs.

**Brain areas**	**MNI coordinates**			**BA**	**Number of voxels**	***T*** **-value**
	**X**	**Y**	**Z**			
Frontal_Sup_R	33	60	12	4	119	−4.3379
Postcentral_R	15	−48	72	58	82	−4.7874

*The statistical threshold was set at P < 0.05 for multiple comparisons with a Gaussian random field, Voxel-level AlphaSim was set at P < 0.01 and rectified for cluster sizes >40 voxels*.

*PerAF, percent amplitude of fluctuation; HCs', healthy controls; Frontal_Sup_R, right dorsolateral superior frontal gyrus; Postcentral_R, right posterior central gyrus*.

### ROC Curve Analysis

**Figure 2:**
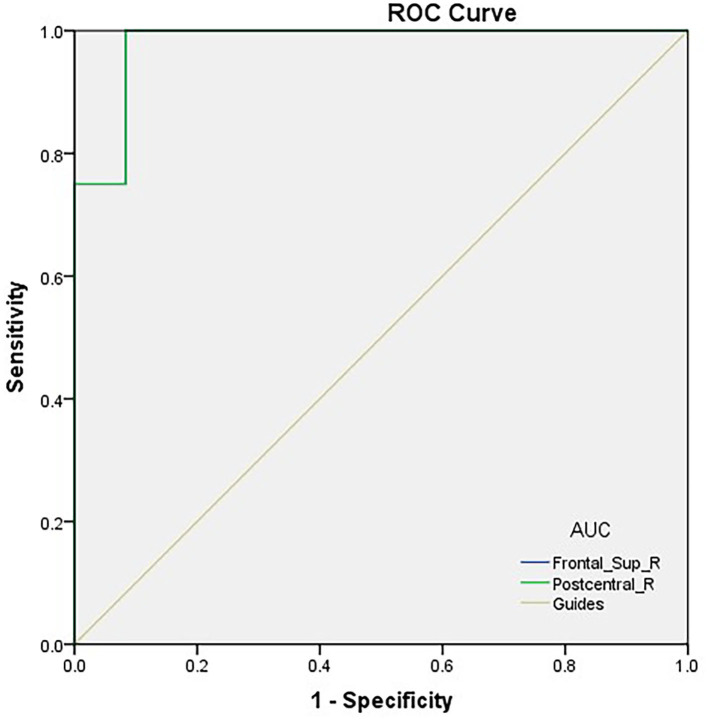
ROC curve analysis of the PerAF values for altered brain regions. ROC curve analysis of the PerAF values for altered brain regions. The area under the ROC curve of the right dorsolateral superior frontal gyrus was 0.979 (*P* < 0.0001; 95% CI: 0.931–1.000); the area under the ROC curve of the right posterior central gyrus was 0.979 (*P* < 0.0001; 95% CI: 0.931–1.000). ROC, receiver operating characteristic; PerAF, percent amplitude of fluctuation; AUC, area under the curve.

We hypothesized that distinctions in PerAF values could be an underlying helpful diagnostic marker to differentiate the TA group from HCs. ROC curve analysis was utilized to test this hypothesis. Average PerAF values were collected from different regions of the brain and analyzed. The accuracy was deemed low if the area under the curve (AUC) was 0.5–0.7, but high when the AUC was 0.7–0.9. The AUC values of PerAF in the two areas were 0.979 in the RDSFG (*P* < 0.0001; 95% CI: 0.931–1.000) and 0.979 in the RPCG (*P* < 0.0001; 95% CI: 0.931–1.000; Figure 2).

### SVM Analysis Results

**Figure 3:**
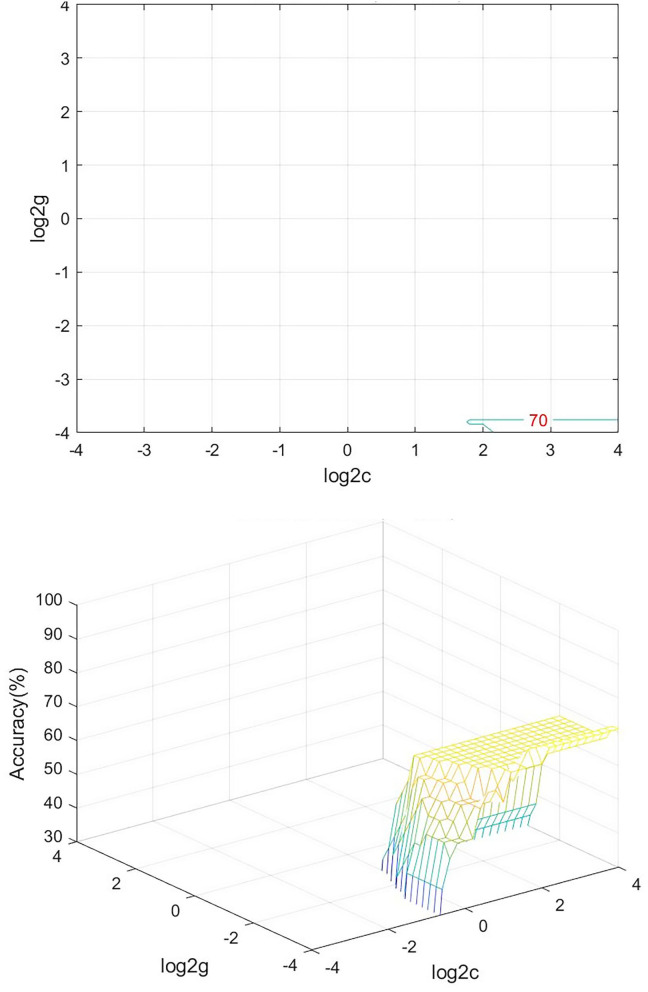
Visualization of SVM results for TA and HC. Left, Visualization of the classification map. Right, 3D view of classification accuracy with best parameters. SVM, support vector machine; TA, toothache; HC, healthy control.

**Figure 4:**
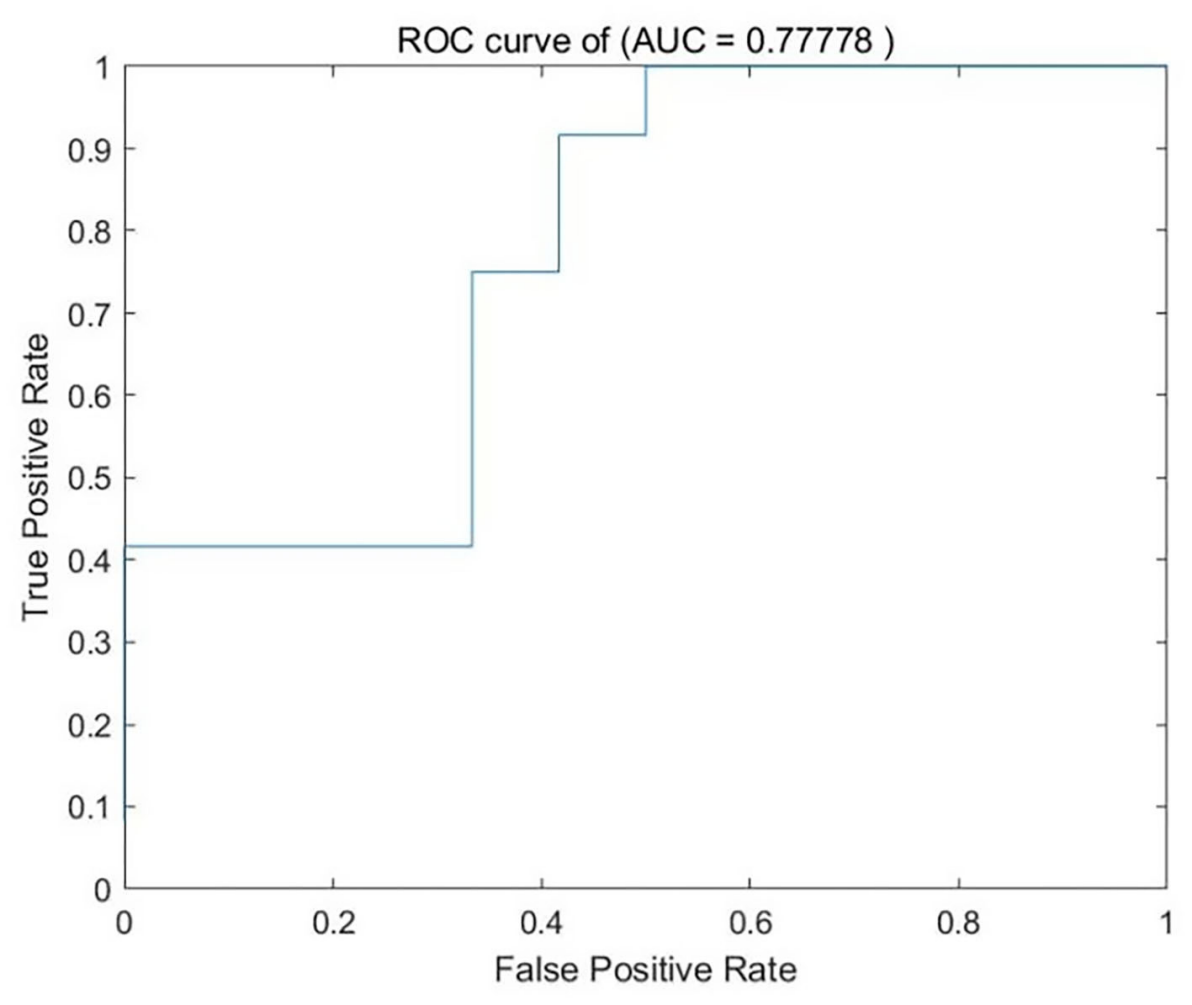
ROC curve of the SVM classifier. SVM, support vector machine; ROC, receiver operating characteristic; AUC, area under the curve.

We analyzed PerAF data from the TA group and HCs. The SVM accuracy results suggested that the PerAF data could be utilized to distinguish the TA group from HCs with a sensitivity of 75.00%, a specificity of 66.67%, and an accuracy of 70.83% (Figure 3). The global accuracy for this combination using the permutation test was 0.6923 (*p* < 0.001), and its ROC curve fitted well (Figure 4).

## Discussion

Since the invention of the electroencephalogram (EEG), humans have begun to explore different neuroimaging tools in a variety of conditions. In recent decades, the characteristics of brain activity in the resting state have become a topic of widespread concern in various medical departments, providing a new perspective for related research on brain functional organization.

**Figure 5:**
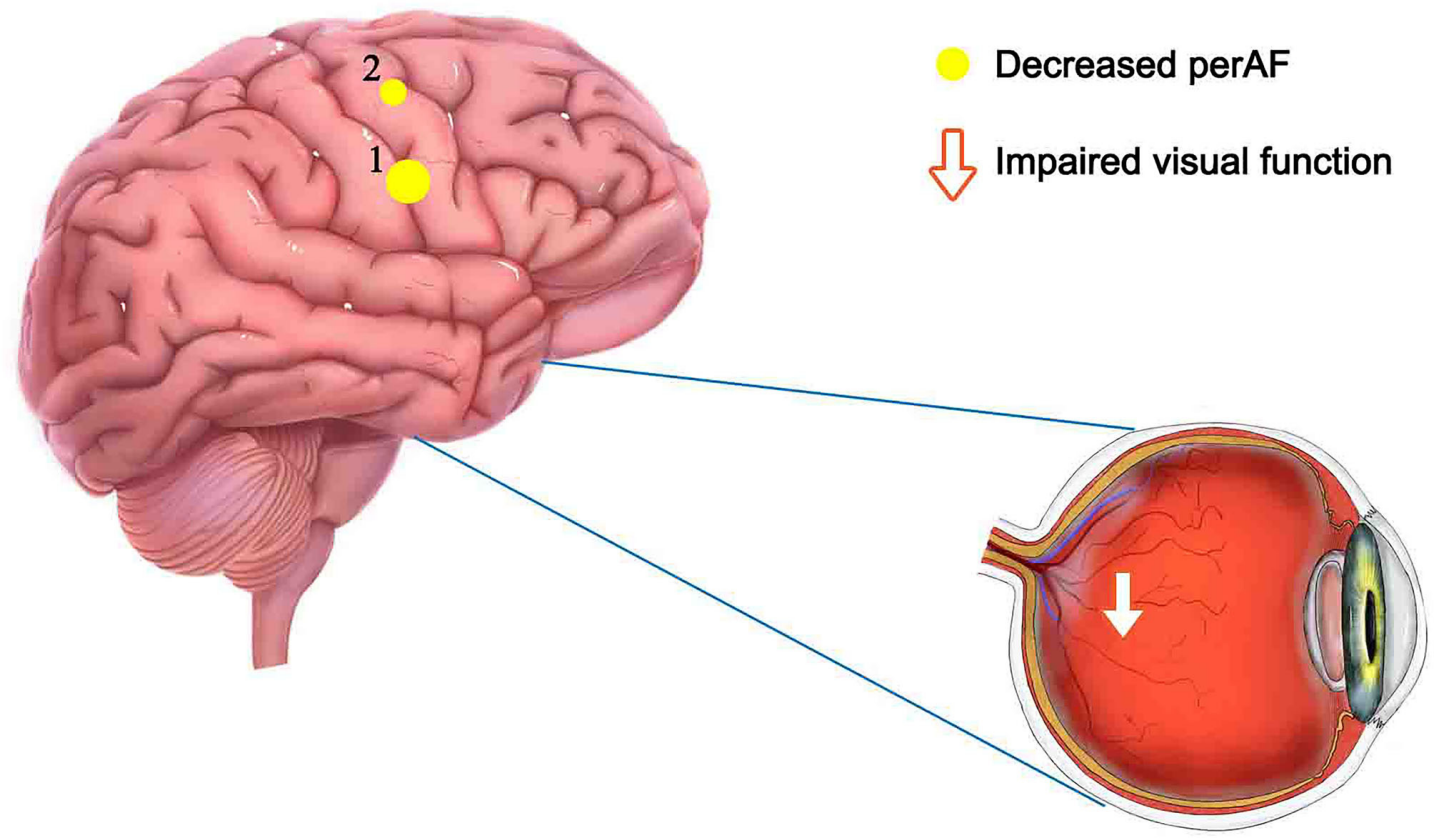
Mean PerAF values for altered brain regions. Compared with HCs, PerAF values in the following regions decreased to varying degrees: 1- right dorsolateral superior frontal gyrus (BA 4, *t* = −4.3395), 2-right posterior central gyrus (BA 58, *t* = −4.7844). PerAF, percent amplitude of fluctuation; HCs, healthy controls; BA, Broadman area.

Percent amplitude of fluctuation, a new and relatively reliable technique, has been utilized in studies of patients with ophthalmic and neurogenic diseases (Table 3). In this investigation, we first utilized the PerAF technique to study neural functions and abnormalities in separate cerebrum areas of patients with TA. The results showed that the signal values of PerAF in the RDSFG and right posterior central gyrus of patients with TA were decreased compared with HCs, and may cause visual impairment (Figure 5).

**Table 3:** PreAF applied in ophthalmic and neurogenic diseases.

**Author**	**Year**	**Disease**	**Brain areas**	
			**PerAF increased**	**PerAF decreased**
Yang et al. (15)	2021	Retinal detachment	RFG, LITG	——
Li et al. (16)	2021	Moyamoya disease	LCN	RMSFG,LPG
Yu et al. (17)	2021	Neovascular glaucoma	LITG	RACG, RSOG, LSFG
Yu et al. (18)	2019	Mild cognitive impairment	LP	——

*PerAF, percent amplitude of fluctuation; RFG, right fusiform gyrus; LITG, left inferior temporal gyrus; LCN, left caudate nucleus; RMSFG, right medial superior frontal gyrus; LPG, left precentral gyrus; LITG, left inferior temporal gyrus; RACG, right anterior cingulate and paracingulate gyrus, RSOG, right superior occipital gyrus; LSFG, left superior frontal gyrus; LP, left parahippocampus*.

The postcentral gyrus, situated in the lateral parietal lobe of the brain, is known as the primary somatosensory cortex (S1) and acts as a crucial area for touch and nociception (19–21). Pain perception is a multi-dimensional experience, including sensory discrimination, emotional motivation, and cognitive evaluation components (22). Downregulation of pain involves cortical and subcortical brain structures, including the dorsolateral prefrontal cortices (DLPFC) and S1. A study of pain induced by thermal stimuli has shown that S1 tissue is typically activated when such incentives are present (23). Previous studies have shown that S1 activity is involved in many pain-related disorders (20, 24, 25). Baciu et al. (24) revealed similar S1 activation in patients with rectal pain, and Binkofski and colleagues (20) described painful esophageal dilation resulting in a marked pattern of brain activation of S1. In another study, somatosensory impairment caused by decreased neural activity in the postcentral gyrus is an early symptom of Parkinson's disease (25).

A previous study has suggested that the DLPFC is often concerned with cognitive and attentive management of algetic stimulus (26). Therefore, a decreased functional correlation between the right S1 and the DLPFC may be associated with altered attentional status to noxious stimuli from T1 to T2. This may explain the simultaneous decline in PerAF values of the RPCG and RDSFG in patients with TA.

Damage to the RPCG results in somatosensory impairment, but experiments have shown that the information processed by the early S1 can distinguish familiar visual objects. This effect was only discovered for familiar objects. This is because correlations from vision to early S1 transmission of content-specific messages about familiar objects only relied on visual facades (27). In another study, when the lesions in the right S1 appeared alone, visual exploration and sensitivity were deviated from the midline and mainly manifested as spatial asymmetry when distinguishing the same target on the retina. This suggested that S1 plays a role in spatial attention allocation, demonstrating a novel role for S1 in visuospatial attention (28). The superior colliculus (SC) is a visual reflex center. The deep layers of SC receive input from S1 (29), and the S1 injury may also cause SC reflex disorders. Therefore, damage to S1 associated with a TA may cause visual impairment.

Specific dystonia is a motion disease that involves abnormal postures of the body. Some regression analysis studies discovered that S1 was markedly connected with variability in fundamental frequency signals related to dystonic symptoms and that somatic assessment portrayed essential distinctions in the right somatosensory cortex (30, 31). Researchers used fMRI data to assess brain activity and physical therapy. The results revealed that the somatosensory representation of S1 was significantly different between dystonic patients and HCs (32). This difference involves the gating system by which the S1 can turn off movement-related sensory input. Such gating systems can filter out redundant inputs, reduce irrelevant information, and play an essential role in organized movement (33, 34). Patients with TA may be at risk of dystonia due to an impaired sensory cortical gating system.

Patients suffering from obsessive-compulsive disorder (OCD) often use repetitive behaviors to relieve anxiety, including touching, counting, and repeated washing. A recent study found an increase in S1 gray matter volume in patients with OCD (35). The manifestations of patients with OCD were similar to sensory-related impulses observed in mental disorders and the impulses were associated with activation of the S1 and insula (36). Motor areas were also regularly connected with heightened sensory impulses and a lack of significant motion, indicating a crucial connection between sensory and action centers in the cerebral cortex that is particularly prominent in disorders such as OCD. Neurologically, schizophrenia severity has been positively correlated with activation in the bilateral somatosensory cortex (postcentral gyrus). Recent findings have shown a connection between somatosensory activation and the severity of OCD, suggesting that the somatosensory areas of OCD patients are functionally and structurally abnormal (37).

In the superior frontal gyrus, researchers observed increased activation associated with various attentional control tasks, including inhibitory control, conflict resolution, and visuospatial interference suppression (38). In addition to interference inhibition, the results of a study suggested that the right DLPFC is involved in adaptive cognitive control, and this brain area participates in cognitive control processing by shielding task-irrelevant interference (39). Corresponding evidence has come from patients with right DLPFC injury who had difficulty developing strategies for coping with interference (40). This finding suggests that TA-induced impairment of the RDSFG may lead to recurrent inattention and cognitive control impairments that are easily disturbed.

In a study aimed at identifying structural variations in the cerebrum areas of sufferers with vestibular migraines (41), a significant increase in the volume of the superior frontal gyrus was found. Some of these cerebrum areas with increased gray matter volume were involved in evaluating pain and were closely related to emotion and anxiety. In another study, the excitability of the superior frontal gyrus was decreased significantly and was significantly correlated with psychological conditions such as depression (42). Additionally, a decrease in the functional connection of the left medial superior frontal gyrus in patients with an untreated first episode of severe depression was noted in a separate study (43). Further studies described motivational deficits in many psychiatric disorders, including major depressive disorder (MDD). For example, patients with Alzheimer's disease and Parkinson's disease are characterized by cognitive decline associated with the frontal cortico-subcortical network, including the superior frontal gyrus, anterior cingulate gyrus, and ventral striatum (44). More precisely, impaired control of subcortical (inferior) structures (amygdala) by cortical (top) structures, such as the DLPFC and ACC, is considered to be the cause of negative emotions in MDD (45). In one study, researchers captured the role of brain regions that coordinate intra-hemispheric and inter-hemispheric correspondence. The results indicated that the bilateral superior frontal gyrus was related to MDD-related reductions. Damaged functional correlations in this region have been frequently reported in a previous MDD study (46). The results further suggested that the damaged functional connectivity involved the ipsilateral hemisphere and the contralateral hemisphere, so the bilateral superior frontal gyrus is considered a critical functional disconnection node in MDD (47). Therefore, the decreased PerAF value of RDSFG in patients with TA may lead to emotional disturbances, cognitive impairment, and even the onset of MDDs.

People with anxiety disorders exhibit a wide range of symptoms, such as psychological experiences and feelings of excessive worry, as well as nervousness, unmanageable worry, and panic attacks. Anxiety disorders may involve many brain areas (48, 49). In one study, researchers tested the role of the DLPFC in anxiety manifestations with 10 Hz repetitive transcranial magnetic stimulation (rTMS). The findings showed that rTMS in the right DLPFC was associated with increased anxiety, suggesting a mechanical connection between right DLPFC function and anxiety manifestations (50). Another study has also shown that the activity in the DLPFC during threatening situations was negatively correlated with anxiety (51), and activation of the DLPFC reduced anxiety-enhanced startle. These findings suggest that promoting DLPFC activity can reduce anxiety and damage to the DLPFC may lead to symptoms of anxiety in patients.

Dynamic and flexible cognition is one of the fundamental key traits of humans and it manifests as bistable or multi-stable perceptions. Clinically, visual bistable perception disorder is more common. The DLPFC is thought to be involved in such spontaneous perceptual transitions (52, 53). A recent study showed that the DLPFC's causal role in spontaneous perceptual reasoning was behaviorally detectable when tracking brain state dynamics that underpinned this perceptual fluctuation, and its activation enhanced functional integration between frontal and intermediate states (54). Therefore, damage to the GDSFG may cause disturbances of bistable perception, which lead to disturbances in the patient's perception ability.

As mentioned above, OCD is a disabling disorder characterized by recurring persistent urges and repetitive behaviors (35, 55). Evidence from structural neuroimaging studies have highlighted the pivotal character of the DLPFC in the pathology of OCD. Compared with healthy controls, DLPFC volume and thickness were reduced in OCD patients (56). In a resting-state functional connectivity study, DLPFC-related dysconnectivity was recognized in OCD patient ROI-restricted analyses (57). A recent study utilized rs-fMRI to investigate the neural prioritization of the DLPFC in sufferers of OCD. They discovered abnormalities in the DLPFC-right orbitofrontal cortex circuit, DLPFC-inferior temporal gyri circuit, and DLPFC cerebellar circuit, verifying neural circuit abnormalities in an OCD patient alternative model (58). Therefore, DLPFC damage related to TA may cause symptoms of OCD in patients.

We found a close connection between the RDSFG and the RPCG. The decreased association between them may be responsible for the painful sensation of TA. Furthermore, damage to both brain regions may lead to complications, such as OCD and cognitive impairment. These findings have certain implications for the mechanism and neuropathological factors of TA and are helpful for the treatment of subsequent complications of TA. Using the ALFF method to study neural activity of patients with TA (59) showed that the activity of the posterior central gyrus was significantly improved, thus indicating that the PerAF method has a good universality.

## Conclusion

However, there are some limitations in our study. First, the sample size of this study was small. In order to acquire more representative results, we need to enlarge the sample size in subsequent studies. Second, the inclusion criteria for patients with TA were not strict, and acute and chronic TA were not further distinguished and odontogenic and non-odontogenic pain were not explicitly classified. We discovered that PerAF signal values in the RDSFG and RPCG were markedly lower in patients with TA than in the HCs. These variations may elevate the risk of relevant diseases related to brain dysfunction (Table 4). Despite the abovementioned limitations of our study, the potential pathogenesis of TA can still be considered as connected with abnormalities in specific brain areas. It is useful to comprehend pathological mechanisms and relevant diseases of patients with TA. In addition, due to the high sensitivity and specificity of ROC curves and SVM results, alternations in PerAF values in specific cerebrum areas can be utilized as one of the effective indicators to examine pain-related diseases.

**Table 4:** Brain regions and potential effects.

**Brain regions**	**Experimental results**	**Brain functions**	**Expected results**
Right dorsolateral superior frontal gyrus	TA < HC	Primary somatosensory, tactile and visual processing	Unbearable pain, early Parkinson's disease, depression, visual impairment
Right posterior central gyrus	TA < HC	Sensory processing, functional connectivity	Pain leads to enlargement of brain area, severe depression and obsessive-compulsive disorder

*TA, toothache; HCs, healthy controls*.

## Data Availability Statement

The original contributions presented in the study are included in the article/supplementary material, further inquiries can be directed to the corresponding author/s.

## Ethics Statement

The studies involving human participants were reviewed and approved by the Medicinal Morality Council of Nanchang University's First Affiliated Hospital. The patients/participants provided their written informed consent to participate in this study.

## Author Contributions

All authors listed have made a substantial, direct, and intellectual contribution to the work and approved it for publication.

## Funding

Key Research Foundation of Jiangxi Province (20203BBG73059, 20181BBG70004, and 20202BBGL73122); Excellent Talents Development Project of Jiangxi Province (20192BCBL23020); Natural Science Foundation of Jiangxi Province (20181BAB205034).

## Conflict of Interest

The authors declare that the research was conducted in the absence of any commercial or financial relationships that could be construed as a potential conflict of interest.

## Publisher's Note

All claims expressed in this article are solely those of the authors and do not necessarily represent those of their affiliated organizations, or those of the publisher, the editors and the reviewers. Any product that may be evaluated in this article, or claim that may be made by its manufacturer, is not guaranteed or endorsed by the publisher.
